# A risk signature of necroptosis-related lncRNA to predict prognosis and probe molecular characteristics for male with bladder cancer

**DOI:** 10.1097/MD.0000000000033664

**Published:** 2023-05-05

**Authors:** Yuzhou Jin, Jiacheng Li, Chenhao Tang, Kangwei He, Donggang Shan, Shenze Yan, Gang Deng

**Affiliations:** a Zhejiang Chinese Medical University, Hangzhou, China; b Hangzhou First People’s Hospital, Hangzhou, China.

**Keywords:** bladder cancer (BC), long non-coding RNA (lncRNA), molecular characteristics, necroptosis, prognostic prediction, risk signature

## Abstract

Bladder cancer (BC) is a frequently diagnosed cancer with high mortality. Male patients have a higher risk of developing BC than female patients. As a type of caspase-independent cell death, necroptosis plays a significant role in the occurrence and progression of BC. The aberrant function of long non-coding RNAs (lncRNAs) plays an indispensable role in GI. However, the relationship between lncRNA and necroptosis in male patients with BC remains unclear. The clinical information and RNA-sequencing profiles of all BC patients were retrieved from The Cancer Genome Atlas Program. A total of 300 male participants were selected for the study. We conducted to identify the necroptosis-related lncRNAs (NRLs) by Pearson correlation analysis. Subsequently, least absolute shrinkage and selection operator Cox regression were conducted to establish a risk signature with overall survival-related NRLs in the training set and to validate it in the testing set. Finally, we verified the effectiveness of the 15-NRLs signature in prognostic prediction and therapy via survival analysis, receiver operating characteristic curve analysis, and Cox regression. Furthermore, we analyzed the correlation between the signature risk score and pathway enrichment analysis, immune cell infiltration, anticancer drug sensitivity, and somatic gene mutations. We developed 15-NRLs (AC009974.1, AC140118.2, LINC00323, LINC02872, PCAT19, AC017104.1, AC134312.5, AC147067.2, AL139351.1, AL355922.1, LINC00844, AC069503.1, AP003721.1, DUBR, LINC02863) signature, and divided patients into a high-risk group and low-risk group through the median risk score. Kaplan–Meier and receiver operating characteristic curves showed that the prognosis prediction had satisfactory accuracy. Cox regression analysis indicated that the 15-NRLs signature was a risk factor independent of various clinical parameters. Additionally, immune cell infiltration, half-maximal inhibitory concentration, and somatic gene mutations differed significantly among different risk subsets, implying that the signature could assess the clinical efficacy of chemotherapy and immunotherapy. This 15-NRLs risk signature may be helpful in assessing the prognosis and molecular features of male patients with BC and improve treatment modalities, thus can be further applied clinically.

## 1. Introduction

Bladder cancer (BC) is the ninth most common malignant disease and the thirteenth most common cause of cancer death worldwide, especially muscle-invasive BC, which usually forms micro-metastases and is unfavorable to patient prognosis.^[1,2]^ Interestingly, although BC is a non-gender-specific cancer, it has been found that men are 3 to 4 times more common to be diagnosed with BC than women.^[3,4]^

Necroptosis is the first form of programmed necrosis described with a prominent role in multiple physiological and pathological conditions.^[5–7]^ The core of the necroptosis machinery is constituted by RIPK1, RIPK3, and MLKL.^[8]^ The exact effect of necroptosis on tumors is complex and controversial.^[8,9]^ Indeed, several studies suggest its involvement as a cancer suppressor that participates in cancer inhibition by activating markers involved in regulated necrosis pathways, while others as cancer promoters by inhibition of tumor immunity and activation of inflammation.^[10,11]^

Long non-coding RNA (lncRNA) is a kind of cellular transcript that is longer than 200 nt in length and does not code for proteins, which mainly functions in transcription regulation, nuclear domain organization, and protein or RNA molecules regulation.^[12]^ Accumulating evidence suggests that lncRNA can act as an oncogene or tumor suppressor to participate in the occurrence and development of cancers.^[13,14]^ Researchers have clarified that lncRNA can participate in the occurrence and development of cancer through necroptosis.^[15–17]^

In this study, we obtained lncRNA data and clinical information related to BC from The Cancer Genome Atlas (TCGA) database and constructed a necroptosis-related lncRNAs (NRLs) based risk signature to explore its role in predicting the prognosis of BC. We assume that this risk signature has the ability to predict the prognosis of male patients with BC. The content of our study is to evaluate and verify the prognostic ability and independent prognostic value of risk signature.

## 2. Materials and methods

### 2.1. Data collection

The clinical information and RNA-sequencing (RNA-seq) profile associated with all BC patients were retrieved from TCGA (https://portal.gdc.cancer.gov/) on September 13, 2022. For subsequent analysis, transcriptome profiles were processed by log2. A total of 300 males with BC were included in the current study. The exclusion criteria were a negative histological diagnosis of BC, the presence of a malignancy other than BC, lack of complete clinical data, and female patients. In each sample, the data of the transcriptome profile contains the expression level of detected lncRNAs in each sample. From the clinical information, we selected age, gender, follow-up time, survival status, smoking status, pathological stage, and TNM stage for analyses. Necroptosis-related genes (NRGs) were acquired by searching the Kyoto Encyclopedia of Genes and Genomes (https://www.kegg.jp/ accessed on September 9, 2022) and the Human Gene Database (https://www.genecards.org/ accessed on September 9, 2022). In addition, the mutation data of BC samples were downloaded in MAF format from the TCGA database. No approval from the ethics committee was needed because all the information was acquired from the TCGA database.

### 2.2. Identification of NRLs in BC

Firstly, differential expression analysis was performed for NRGs between BC tissues and normal bladder tissues. Then, we identified lncRNAs from the TCGA-BC RNA-seq data, and the co-expression relationships between differentially expressed NRGs and all lncRNAs in BC samples were examined by Pearson correlation analysis. |Coefficient| > 0.3 and *P* value < 0.01 were considered as the cutoff.

### 2.3. Establishment of the NRLs-mRNA co-expression and protein-protein interaction (PPI) network

In order to prove the mutually regulated connection between the NRLs and the corresponding target mRNAs, we use Cytoscape (version 3.9.0, www.cytoscape.org) to visualize the lncRNA-mRNA network. In addition, the NRGs regulated by candidate lncRNAs were uploaded to the string (version 11.5, www.string-db.org) website to construct the PPI network.

### 2.4. Identification of overall survival (OS)-related NRLs

In all NRLs, OS-related lncRNAs play a key role in the onset, progression, and prognosis of tumors. We performed time-dependent univariate Cox regression with the ezcox package (http://www.bioconductor.org/packages/release/bioc/html/ezcox.html) in R (version 4.1.1, https://www.r-project.org/) to screen NRLs related to OS. The cutoff criterion for statistically significant correlations with OS was decided as *P* < .05.

### 2.5. Construction and verification of the NRLs-based risk signature for prognosis prediction

We divided all tumor cases into a training set and a testing set to develop and validate risk signatures. The training set accounted for 60%, and the testing set accounted for 40% (180 cases of training set and 120 cases of testing set). We performed a chi-square test on the clinical data from the 2 sets to ensure that the training set and testing set were unbiased. Finally, each BC patient’s survival risk score (RS) was calculated based on the standardized expression levels of NRLs and the corresponding regression coefficients derived from the least absolute shrinkage and selection operator (LASSO) regression analysis. The calculation is as follows:


$$\begin{array}{l} \begin{array}{lll} Risk\;score & = & expression\;level\;of\;lncRNA1*\beta 1\\ & & +\;expression\;level\;of\;lncRNA2*\beta 2\\ & & +\ldots +\;expression\;level\;of\;lncRNAn*\beta n, \end{array} \end{array}$$

where RS is an indicator to measure the prognosis of BC patients, and β is the regression coefficient of each variable. The median RS of the training set was utilized as the demarcation point to categorize all included BC samples into the low-risk or high-risk subsets.

### 2.6. Verification of the effectiveness in prognosis predicting the risk signature

After risk signatures have been developed, their reliability and robustness in estimating prognostic outcomes must be assessed. The validity of the signature is verified from the following 3 aspects: Kaplan–Meier curves were adopted to contrast the OS of the high-risk and low-risk subsets in the training and testing sets using the survival package. The time-dependent receiver operating characteristic (ROC) curves were used to evaluate survival prediction, and areas under the ROC curve were calculated to assess the predictive accuracy and specificity of the NRLs signature. Univariate and multivariate Cox regression analyses on the whole dataset were performed to assess whether the risk signature was an independent prognostic predictor of survival in BC patients.

### 2.7. Pathway enrichment analysis

In order to clarify the differences in enriched pathways between the low-risk and high-risk subsets, the clusterProfiler package (http://www.bioconductor.org/packages/release/bioc/html/clusterProfiler.html) in R was utilized to carry out the Gene Set Enrichment Analysis.

### 2.8. Immune cell infiltration analysis

The components of immune cells and stromal cells in the tumor microenvironment (TME) of each BC sample were calculated to verify the differences in microenvironment features between low-risk or high-risk subsets using the ESTIMATE package (http://www.bioconductor.org/packages/release/bioc/html/ESTIMATE.html). The relative proportions of 22 types of human immune cells were extracted by the CIBERSORT package (http://www.bioconductor.org/packages/release/bioc/html/CIBERSORT.html), and the correlation between the risk signature and immune cell characteristics was revealed.

### 2.9. Immune checkpoint genes analysis

To explore the therapeutic effect of immune checkpoint gene blockade, we also calculated the expression of key immune checkpoint genes, including PDCD1, PD-L1, and CTLA-4.

### 2.10. Significance of the NRLs-based signature in chemotherapy and immunotherapy

In order to predict the response of BC patients to chemotherapeutic drugs in 2 different risk subgroups, the “oncoPredict” package (http://www.bioconductor.org/packages/release/bioc/html/oncoPredict.html) was used to assess the half-maximal inhibitory concentration (IC50) of ordinary chemotherapy drugs. Wilcoxon signed-rank test was used to analyze the difference in IC50 values between the high-risk subset and low-risk subset.

### 2.11. Analysis of the mutation profiles between low-risk and high-risk subsets

Information on somatic gene mutations of BC patients was employed to investigate the correlations between tumor mutation burden (TMB) and RS. This hypothesis was confirmed by comparing the mutation spectrum of the 2 low-risk and high-risk subsets with maftools package (http://www.bioconductor.org/packages/release/bioc/html/maftools.html) in R.

## 3. Results

### 3.1. Data collection

**Figure 1.**
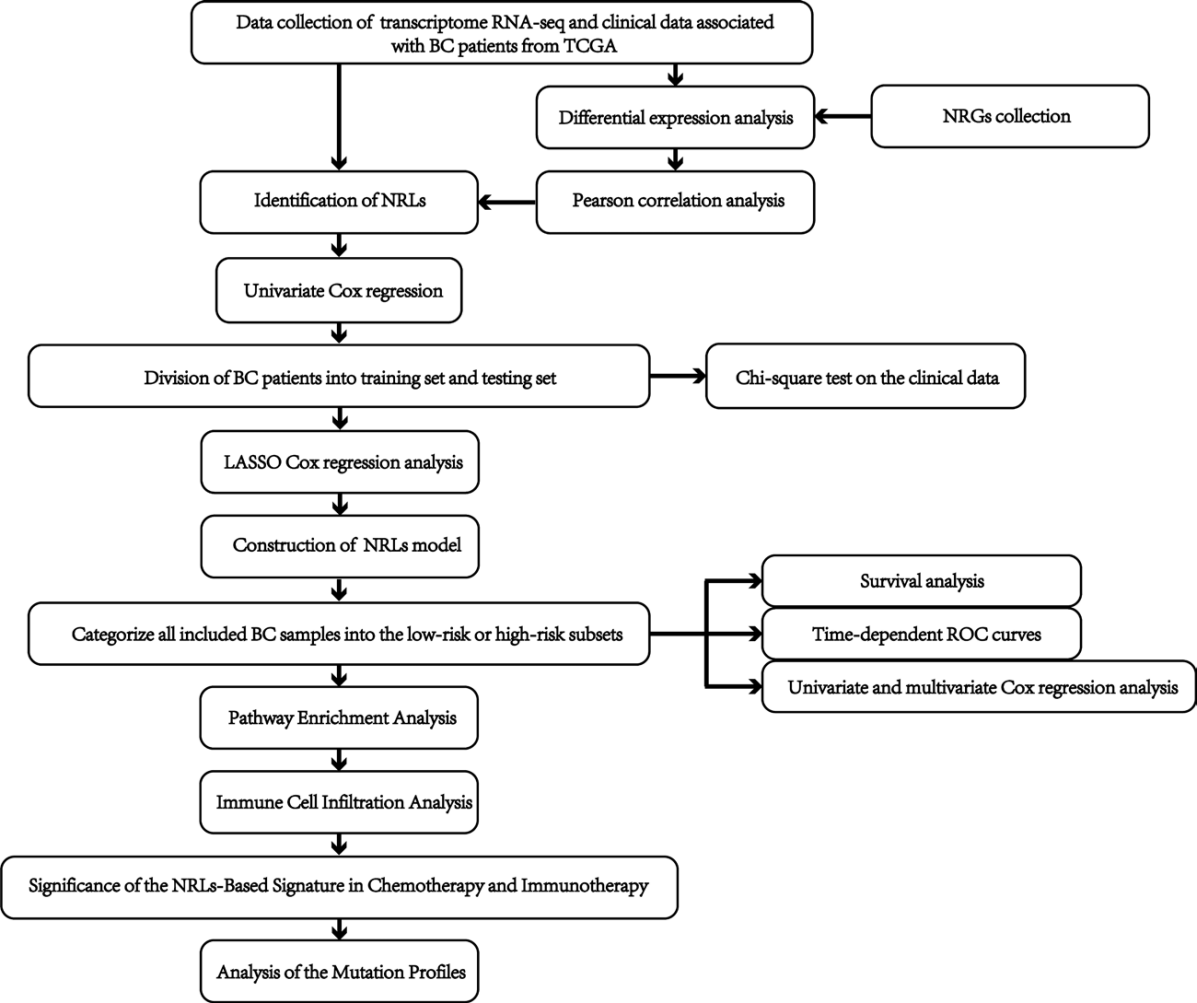
The design of the flow chart and the overall procedures of our study.

Figure 1 shows the flow chart of the research scheme. The transcriptome RNA-seq and clinical data of 432 BC patients were collected from the TCGA database. Three hundred ten male samples were picked out for follow-up analysis, which contained 300 samples of BC tissues and 10 samples of normal bladder tissues. Moreover, 159 NRGs were collected from the Kyoto Encyclopedia of Genes and Genomes (https://www.kegg.jp/ accessed on September 9, 2022) using the keyword “necroptosis”; and 636 NRGs were collected from the Human Gene Database (https://www.genecards.org/ accessed on September 9, 2022) with the keyword “necroptosis gene.”

### 3.2. NRGs differential analysis

**Figure 2.**
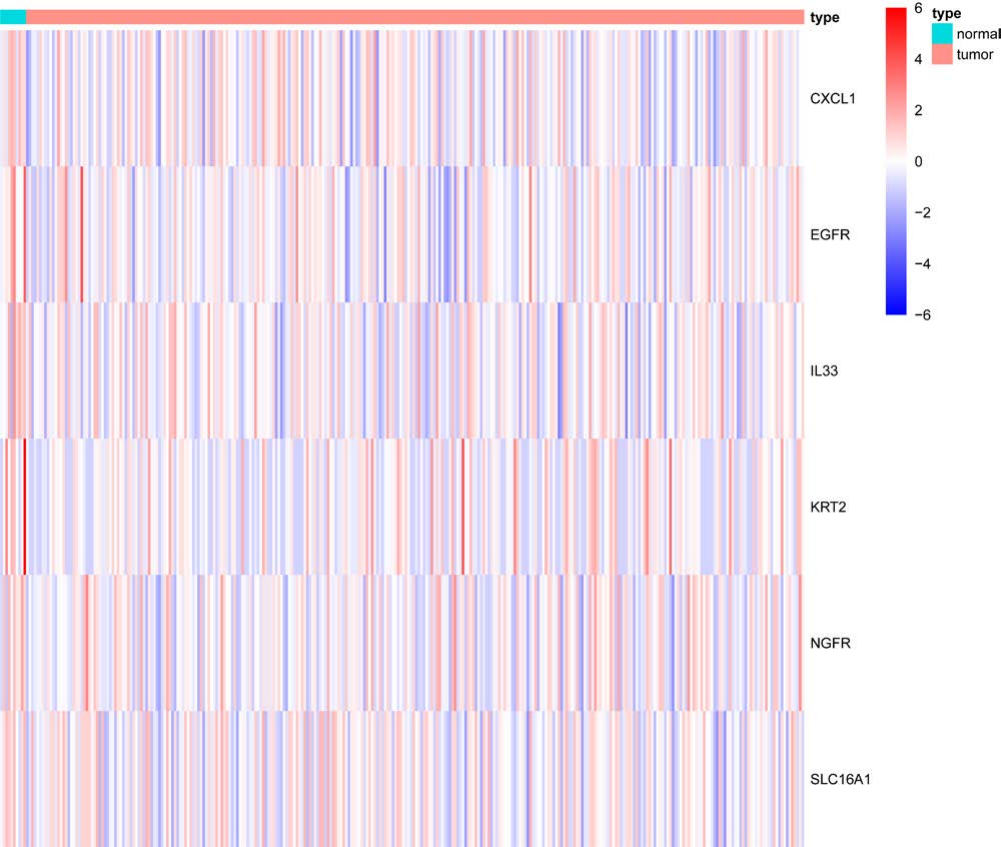
Heatmaps of necroptosis-related genes (NRGs) between bladder cancer tissues and normal bladder tissues. Each cell represents the level of NRG expression in a sample. Red represents high-expression and blue represents low-expression (The expression values were log2 transferred before mapping).

A total of 6 NRGs were differentially expressed between BC tissues and normal bladder tissues, with differentially downregulated (Fig. 2).

### 3.3. Identification of NRLs in BC

**Figure 3.**
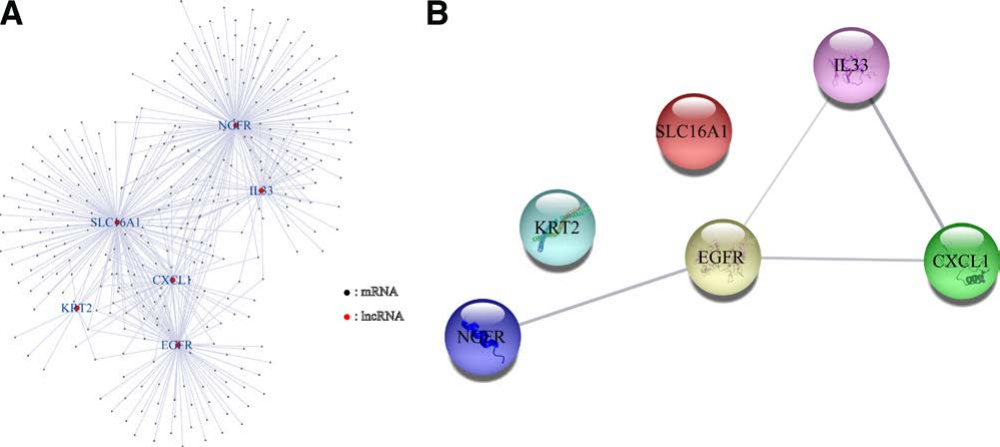
Establishment of lncRNA-mRNA co-expression and the protein-protein interaction (PPI) network. (A) Necroptosis-related lncRNA-mRNA co-expression network diagram. (B) PPI network of necroptosis-related genes. lncRNA = long non-coding RNA.

We first identified 16,876 lncRNAs from the TCGA-BC RNA-seq data, and 1125 lncRNAs were ultimately selected as NRLs using Pearson correlation analysis. An mRNA-lncRNA co-expression network was established to determine the potential effects of NRLs (Fig. 3A). A PPI network was also established to investigate the connection of these NRGs using the STRING database (Fig. 3B).

### 3.4. Construction of the NRLs prognostic signature

**Figure 4.**
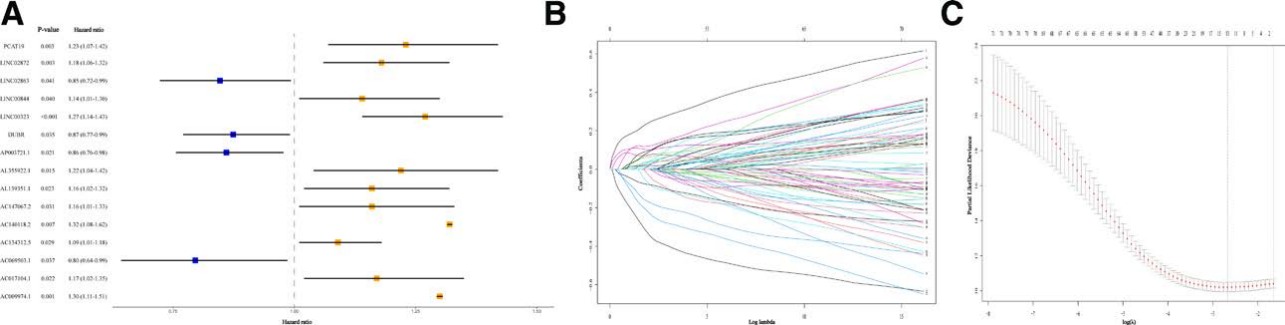
Univariate regression analysis and LASSO regression analysis. (A) The forest plot of univariate Cox regression results of necroptosis-related long non-coding RNAs (NRLs). (B) LASSO coeffificient profifiles of NRLs. (C) The partial likelihood deviance with changing of log(λ). LASSO = least absolute shrinkage and selection operator.


The 300 BC male samples with lncRNA expression data and lncRNA expression data survival information in the TCGA database were used to determine the relationship between NRLs and prognosis. A risk signature was established for the final prognosis prediction. First of all, 71 NRLs associated with OS in BC male patients were screened out by univariate COX proportional hazard regression analysis (*P* < .05, Fig. 4A). Then, the 300 BC samples were randomly allocated into either the training set (n = 180) or the testing set (n = 120) at a ratio of 3:2 (see Supplementary Table S1, Supplemental Digital Content, http://links.lww.com/MD/I903, which illustrates the information of patients in the 2 sets and results of chi-square test.). LASSO Cox regression analysis was conducted using the training set to acquire the lncRNAs with the highest prognostic value via the “glmnet” package (http://www.bioconductor.org/packages/release/bioc/html/glmnet.html) (Fig. 4B and C). Finally, 15 lncRNAs were selected, including 11 risk lncRNAs, namely, AC009974.1, AC140118.2, LINC00323, LINC02872, PCAT19, AC017104.1, AC134312.5, AC147067.2, AL139351.1, AL355922.1, LINC00844, and 4 protective lncRNAs, namely, divide. Based on the expression levels of the 15 lncRNAs and the corresponding weighted coefficients, the risk signature was calculated according to the following formula:

$$\begin{array}{l} \begin{array}{lll} risk\;score & = & 0.1860359095*AC009974.1+\\ & & 0.0363319363*AC140118.2+\\ & & 0.1074923632*LINC00323+\\ & & 0.0328906049*LINC02872+\\ & & 0.0816088895*PCAT19+\\ & & 0.0001294984*AC017104.1-\\ & & 0.2078007600*AC069503.1+\\ & & 0.0081995002*AC134312.5+\\ & & 0.0142401067*AC147067.2+\\ & & 0.1066916565*AL139351.1+\\ & & 0.0239881415*AL355922.1-\\ & & 0.0481047070*AP003721.1-\\ & & 0.1151964849*DUBR+\\ & & 0.0320754831*LINC00844-\\ & & 0.0854706212*LINC02863. \end{array} \end{array}$$

### 3.5. Assessment of the 15-NRLs risk signatures

**Figure 5.**
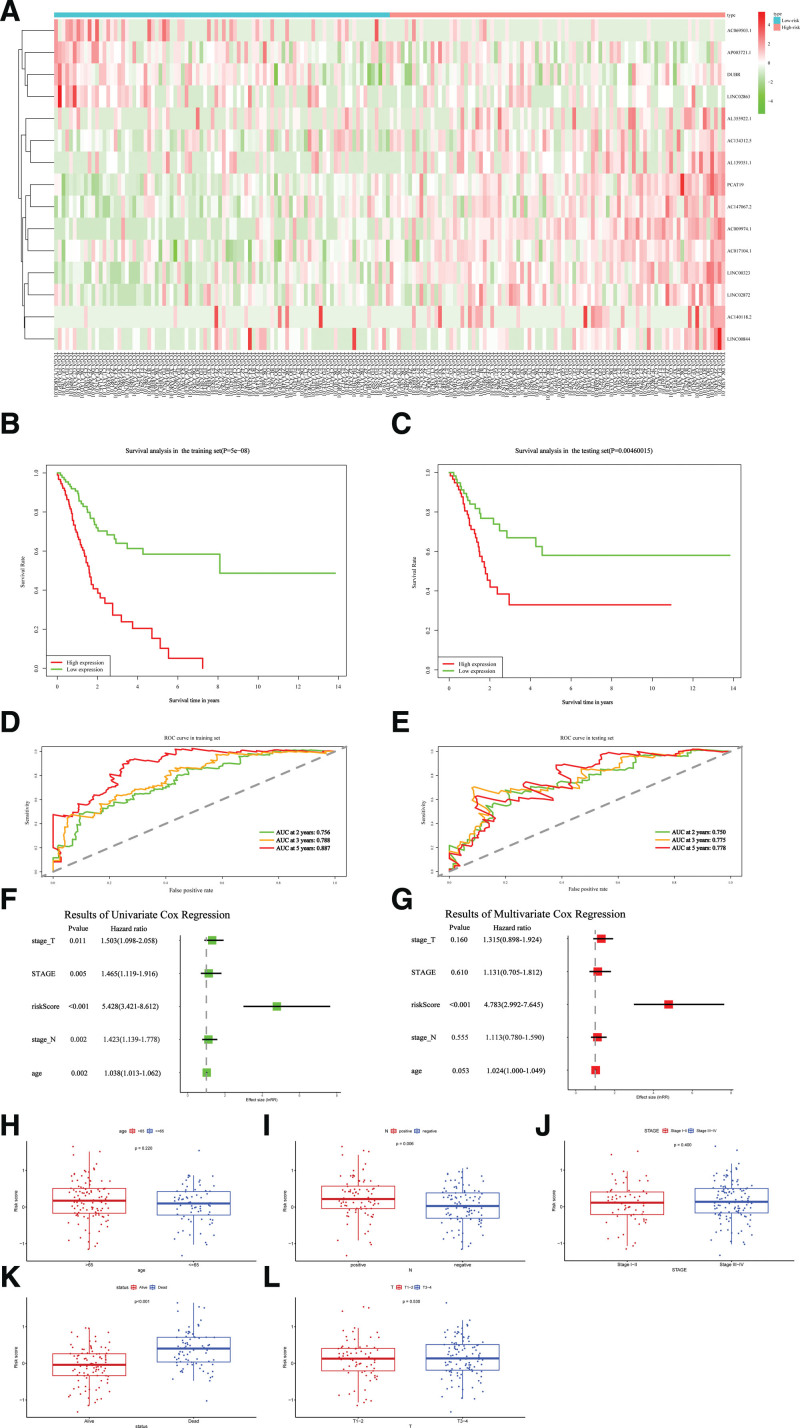
Validation of the prognostic performance of the 15-necroptosis-related long non-coding RNAs (NRLs) signature. (A) The heatmap was drawn to show the expression outlines of the 12 NRLs in the high-risk and low-risk subsets. (B) Survival analysis of training set. (C) Survival analysis of testing set. (D) Receiver operating characteristic (ROC) analysis for evaluating the predictive efficiency at 2-, 3-, and 5-year overall survival (OS) in the training set. (E) ROC analysis for evaluating the predictive efficiency at 2-, 3-, and 5-year OS in the testing set. (H–L) Demonstrated that age (≤65 vs >65), stag, T stages (T1/T2 vs T3/T4), N stages (N0 vs N1/N2/N3), and future state was apparently associated with the risk score.

Firstly, the median RS of the training set was utilized as the cutoff value to assort both the training and testing sets into 2 subsets: low-risk and high-risk. Heatmaps were drawn to show the expression profiles of 15-NRLs in low-risk and high-risk subsets (Fig. 5A). Among them, 11 risk lncRNAs were significantly upregulated and 4 protective lncRNAs were significantly downregulated in high-risk subsets. OS analysis of the training set and testing set showed that patients in the high-risk subset identified by our risk signature had significantly worse outcomes than patients in the low-risk subset (Fig. 5B and C). ROC analysis of the training set and testing set also showed the prediction performance of the risk signature. The areas under the ROC curve values of 2, 3, and 5 years for the training and testing sets reached 0.756, 0.788, 0.887, and 0.750, 0.775, 0.778, respectively (Fig. 5D and E). Univariate and multivariate COX regression analyses on the training sets showed that our risk signature was an independent risk factor for BC patients (Fig. 5F and G). According to the classification of clinical features, we performed RSs for each layer of clinical features separately. According to information from TCGA databases, patients with lymph-node-positive BC had significantly higher RSs in the former compared with patients with lymph-node-negative BC. And future state was apparently associated with the RS. Regarding the future survival status of patients, patients with lower RSs had better long-term survival status (Fig. 5H–L).

### 3.6. Pathway enrichment analysis

**Figure 6.**
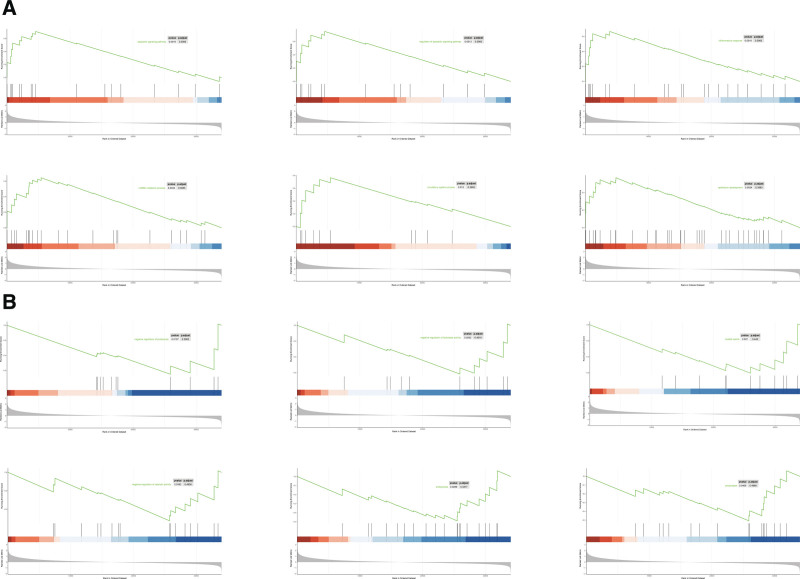
The enriched pathways in high-risk and low-risk subsets obtained using the 15-necroptosis-related long non-coding RNAs (NRLs) signature. (A) Top 6 significantly enriched pathways in the high-risk subset. (B) Top 6 significantly enriched pathways in the low-risk subset.

In order to clarify the differences in enrichment pathway between the low-risk subset and high-risk subset, enrichment analysis was carried out by Gene Set Enrichment Analysis. The results revealed that the significantly enriched in the high-risk subset were negative regulation of proteolysis, negative regulation of hydrolase activity, coated vesicle, negative regulation of catalytic activity, endocytosis, and presynapse (Fig. 6A). Epithelium development, circulatory system process, ncRNA metabolic process, inflammatory response, regulation of apoptotic signaling pathway and apoptotic signaling pathway were significantly enriched in the low-risk subset (Fig. 6B).

### 3.7. The characteristics of TME and immune cell infiltration between the high-risk and low-risk subsets

**Figure 7.**
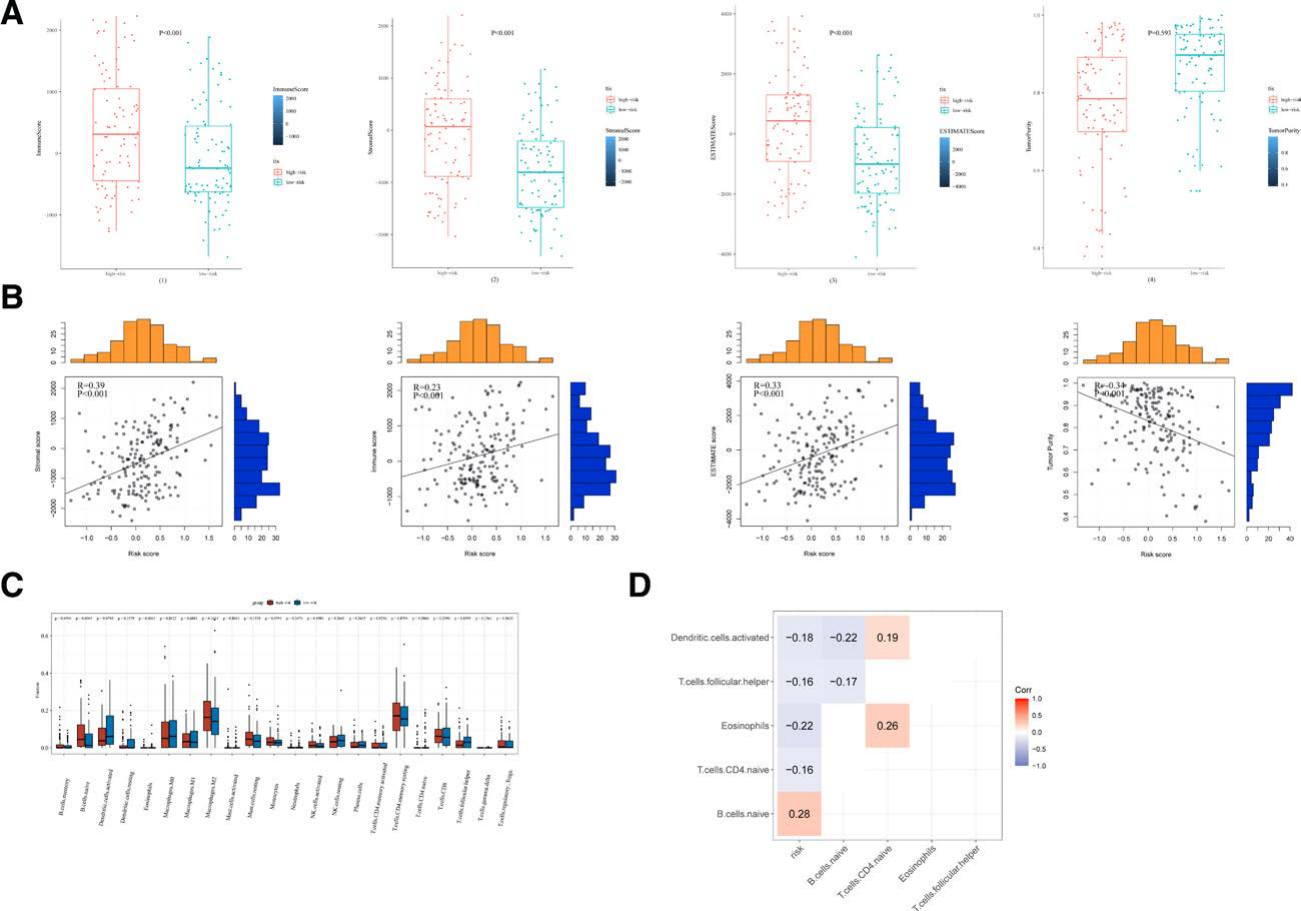
Immune infiltration in the high-risk and low-risk subsets. (A) Comparison of the immune score, stromal score, ESTIMATE score, and tumor purity between the low-risk and high-risk subsets. (B) The relationship between risk score and stromal score, immune score, ESTIMATE score, and tumor purity. (C) Difference analysis of 22 immune cells infiltration between the high-risk and low-risk subsets. (D) Correlation analysis between infiltrating level of B cells naive, T cells CD4 naive, eosinophils, dendritic cells, and activated T cells follicular helper and risk score.

TME was generally composed of tumor cells, stromal cells, and immune cells. TME scores were used to measure the differences in matrix and immune cell permeability between the low-risk subset and high-risk subset. The samples in the high-risk subset had higher immune, interstitial, and evaluation scores and lower levels of tumor purity (Fig. 7A). As shown in Figure 7B, high-RSs were positively associated with higher immune scores, stromal scores, evaluation scores, which were negatively associated with tumor purity. In order to further understand the difference of TME in different risk subsets, the relative expressions of 22 common infiltrating immune cells between the 2 groups were calculated and compared. The results showed that B cells naive was significantly upregulated in high-risk subsets, while eosinophils and T cells gamma delta were significantly upregulated in low-risk subsets (Fig. 7C). It is worth noting that the B cells naive (COR = 0.28) are positively correlated with RSs, while T cells CD4 naive (COR = 0.16), eosinophils (COR = 0.22), Dendritic cells (COR = 0.18), activated T cells follicular helper (COR = 0.16) are negatively correlated with RSs. indicating that part of the reason for the poor prognosis of patients in the high-risk subgroup may be the immune microenvironment (Fig. 7D).

### 3.8. Immune checkpoint genes analysis for the signature

**Figure 8.**
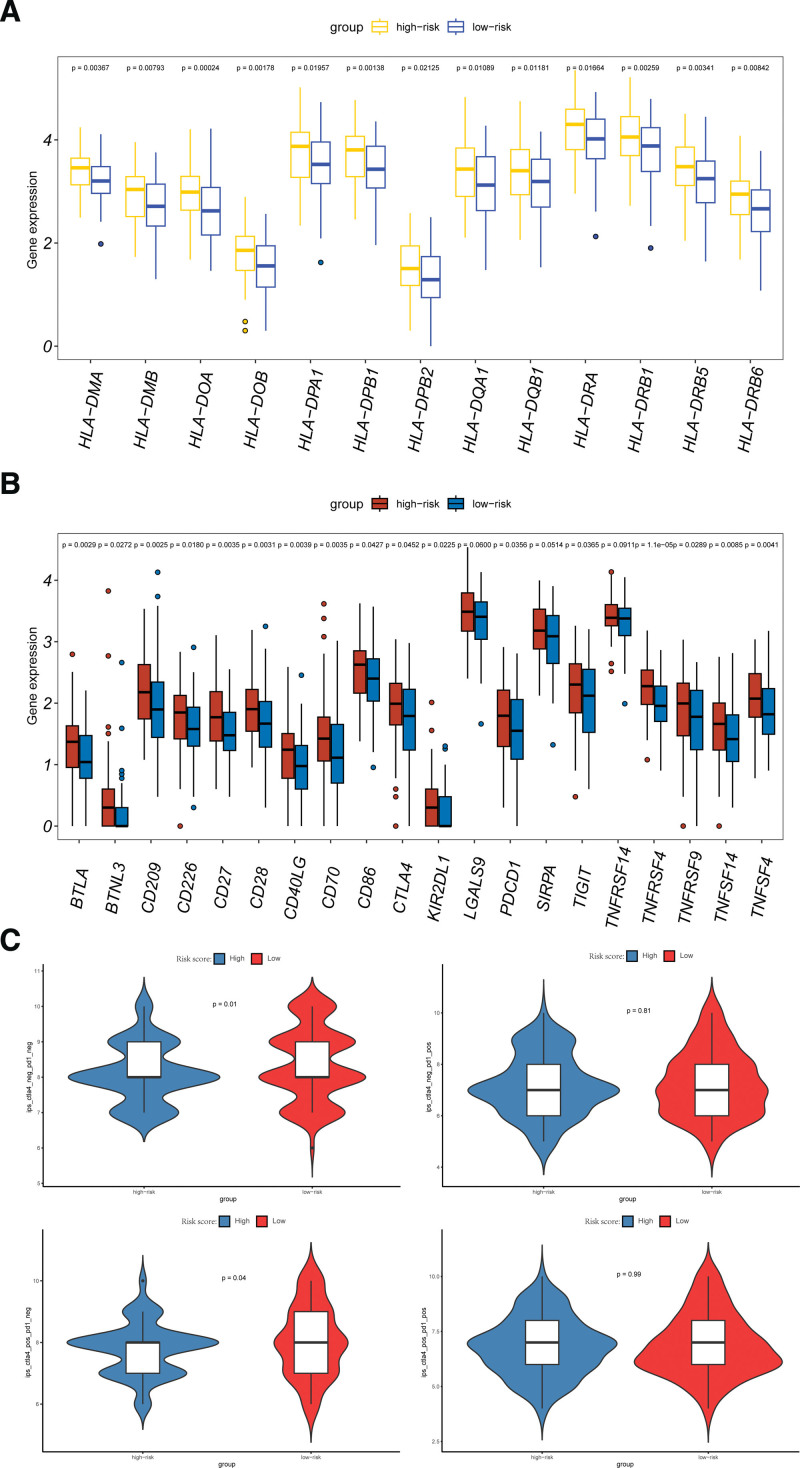
(A) The expression levels of HLA genes in high and low subsets. (B) The expression of key immune checkpoint genes between different subsets. (C) Immunophenoscore (IPS) in different subsets comparisons of the clinical benefit of targeting CTLA-4(+)+PD-1(−), CTLA-4(−)+PD-1(+), CTLA-4(+)+PD-1(+), CTLA-4(−)+PD-1(−).

In the high-risk subset, BTLA, BTNL3, CD209, CD226, CD27, CD28, CD40LG, CD70, CD86, CTLA-4, KIR2DL1, LGALS9, PDCD1, SIRPA, TIGIT, TNFRSF14, TNFRSF4, TNFRSF9, TNFSF14, and TNFSF4CD276 were highly expressed (Fig. 8A). HLA can be divided into class I, class II and class III gene regions, which are mainly involved in antigen presentation on the cell surface and immunomodulation of the body. HLA can activate the immune response by delivering processed antigens to T lymphocytes. It has been shown that high expression of certain molecules in HLA is associated with poor prognosis for some human cancers. As shown in Figure 8B, HLA-DMA, HLA-DMB, HLA-DOA, HLA-DPA1, HLA-DPB1, HLA-DPB2, HLA-DQA1, HLA-DQB1, HLA-DRA1, HLA-DRA1, HLA-DRB1, HLA-DRB5, HLA-DRB6, and other genes were expressed significantly higher in the high-risk subset than in the low-risk subset. We established a correlation between immune checkpoint genes and immunophenoscore based on the expression status of CTLA-4 and PD-1, and we compared the immunophenoscore between the high-risk and low-risk subsets, whose results showed that CTLA-4 gene positivity had significantly different immune responses in the high-risk and low-risk subsets (Fig. 8C).

### 3.9. The importance of the NRLs-based signature in chemotherapy and immunotherapy

**Figure 9.**
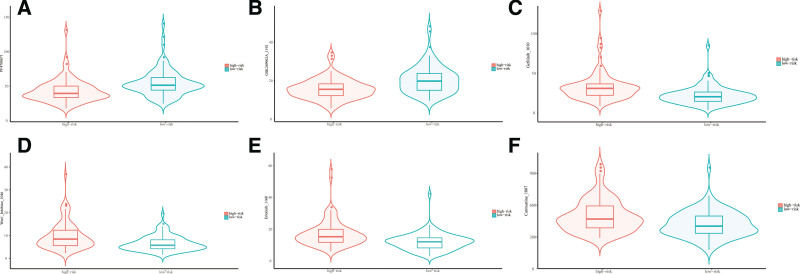
The significance of the necroptosis-related lncRNAs-based signature in chemotherapy and immunotherapy. (A–F) Sensitivity performance of 6 common chemotherapy agents in the high-risk and low-risk subsets. lncRNA = long non-coding RNA.

The 2 risk subsets had a statistically significant difference in their responses to the 15 anticancer drugs. Moreover, patients in the low-risk subset were particularly sensitive to PF-4708671 and GSK269962A, which may be applied to BC patients with a lower RS (Fig. 9A and B). The IC50 values of Gefitinib, Wee1 Inhibitor, Erlotinib, and Carmustine in the high-risk subset were higher, and these 11 drugs may be more applicable for patients with a higher RS based on the 15-NRLs signature (Fig. 9C–F).

### 3.10. The landscape of somatic gene mutations based on the 15-NRLs risk signature

**Figure 10.**
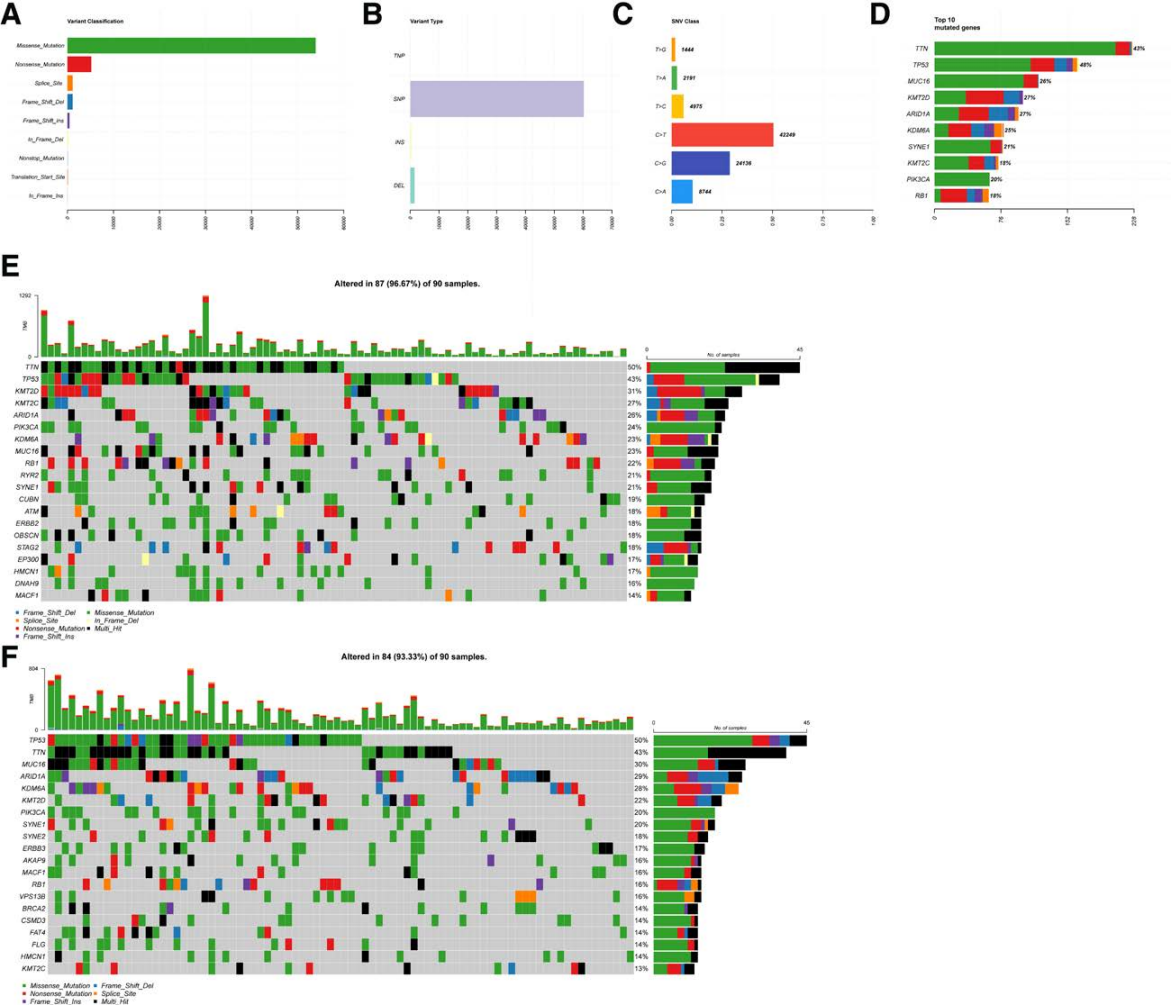
The mutation profile of male patients with bladder cancer (BC). (A) the mutations were classified according to their effects. (B) Classification of mutations by different patterns. (C) The mutations were classified by base transitions. (D) The top 10 frequent mutations in BC patients. (E–F) Mutation profiles of high-risk and low-risk groups based on our risk signature.

Given the important influence of TMB affecting the clinical responses of immune checkpoint inhibitors, we employed the information on somatic gene mutations of BC patients to investigate the correlations between TMB and our risk signature. According to different classified categories, a missense mutation is the most frequent type of mutation in BC (Fig. 10A). The amount of single nucleotide polymorphism is significantly larger than that of insertion or deletion (Fig. 10B). The conversion of cytosine to thymine is the most common base mutation (Fig. 10C). TTN is the most frequently mutated gene in BC, accounting for 43% of the total number of patients with BC (Fig. 10D). The incidence of mutations was lower in the low-risk group than in the high-risk group. Some of the mutated genes common in the high-risk group were rarely observed in the low-risk group, except for individual genes (Fig. 10E and F).

## 4. Discussions

Considerable literatures focus on the effect of necroptosis on BC, but there is a lack of research on the effect of NRLs. Therefore, studying the prognostic value and molecular characteristics of NRLs in BC is of great significance for the diagnosis and treatment of BC patients. In this study, NRLs were obtained from TCGA-BC transcriptional data by Pearson correlation analysis. Then univariate analysis and LASSO regression analysis were used to determine the most valuable NRLs for the prognosis of BC. Finally, 15 lncRNAs were selected to create a 4-lncRNAs risk signature associated with necroptosis. AC009974.1, AC140118.2, LINC00323, LINC02872, PCAT19, AC017104.1, AC134312.5, AC147067.2, AL139351.1, AL355922.1, LINC00844, AC069503.1, AP003721.1, DUBR, and LINC02863 form our risk signatures.

DUBR suppressed the malignant progression of ovarian cancer by downregulating miR-107 to induce SMAC expression and involvement in the XIAP/ caspase 3 signaling pathway.^[18]^ Additionally, DUBR can suppress migration and invasion of lung adenocarcinoma by inhibiting cell oxidative phosphorylation.^[19]^ LINC00844 overexpression significantly repressed the proliferation, migration, and invasion of hepatocellular carcinoma cells by inactivating the mitogen-activated protein kinase signaling pathway.^[20,21]^ LINC00844 was a novel coregulator of the androgen receptor that plays a central role in preventing prostate cancer cell migration and invasion.^[22]^ Furthermore, LINC00844 significantly reduced proliferation and elevated apoptosis of prostate cancer through upregulating GSTP1, which played vital role in cell-cycle regulation.^[23]^ PCAT19 can facilitate MELK-driven glioma tumorigenesis through sponging miR-142-5P.^[24]^ Upregulation of PCAT19 reversed the effects of Honokiol and anisomycin on promoting cell proliferation and inhibiting lung cancer cell apoptosis.^[25]^ Moreover, some studies showed that PCAT19 is implicated in PCa cell growth and tumor progression.^[26–28]^

The nature of TME in the evolution of cancer has changed our understanding of cancer development.^[29,30]^ A wide variety of immune and non-immune cells within the TME infrastructure, together with the factors that they secrete, cooperate to create a chronic inflammatory, immunosuppressive, and protumoral environment.^[31]^ Moreover, TME can not only interact with tumor cells, make tumor cells proliferate, and prevent tumor cell apoptosis and metastasis, but also play an important role in the treatment of tumors.^[32]^ In this study, samples in the high-risk subset had higher immune, interstitial, and evaluation scores and lower levels of tumor purity. Further analysis revealed that the expression of B cells naive in the high-risk subset was significantly upregulated, indicating that these abnormal infiltrating immune cells may be correlated with BC initiation and development. In particular, B cells naive was positively correlated with RS, suggesting that the unfavorable prognosis of patients in the high-risk subset may be partly due to the infiltrated degree of B cells. In addition, the safety and efficacy of PD-1 immune checkpoint inhibitors and CTLA-4 inhibitors have been reported to have been evaluated in several tumor treatments with promising results.^[33–35]^

Chemotherapy and immunotherapy accompanied by surgery have become the primary approaches for BC.^[36]^ In theory, the use of chemotherapy in an adjuvant setting may be optimal after surgery.^[37,38]^ Meanwhile, immunotherapy has demonstrated remarkable success in BC at various stages of the disease.^[39]^ Higher tumor load before surgery or radiotherapy results in a more effective T-cell response.^[40]^ Patients in the low-risk subset may be sensitive to drugs such as Doramapimod and Ribociclib. Some drugs like Gefitinib, Erlotinib, and Carmustinemay be more applicable for patients with a higher RS.

This study had a few limitations. First, relatively few datasets were collected. More independent datasets are thus needed for further validation. Second, more biological experiments are needed to validate and explore the mechanism of action of necroptosis. We are currently conducting clinical trials, but this is a very time-consuming process. We will clinically verify our conclusions in follow-up studies.

In short, this study successfully created a 15-NRLs signature to predict the survival of BC patients. In addition, the study of the molecular features based on 15-NRLs signature was essential to expand new strategies and ideas for the treatment of male patients with BC.

## 5. Conclusions

This 15-NRLs risk signature may be helpful to assess the prognosis and molecular features of male patients with BC and improve treatment modalities, thus can be further applied clinically.

## Author contributions

**Conceptualization:** Yuzhou Jin, Gang Deng.

**Data curation:** Yuzhou Jin, Jiacheng Li.

**Formal analysis:** Yuzhou Jin, Gang Deng.

**Investigation:** Yuzhou Jin.

**Methodology:** Yuzhou Jin, Chenhao Tang.

**Software:** Yuzhou Jin, Shenze Yan.

**Validation:** Yuzhou Jin, Kangwei He.

**Visualization:** Yuzhou Jin, Donggang Shan.

**Writing – original draft:** Yuzhou Jin, Gang Deng.

**Writing – review & editing:** Yuzhou Jin, Gang Deng.

## Supplementary Material


